# Distinct Gene Expression Profiles in Immortalized Human Urothelial Cells Exposed to Inorganic Arsenite and Its Methylated Trivalent Metabolites

**DOI:** 10.1289/ehp.8174

**Published:** 2005-08-17

**Authors:** Pei-Fen Su, Yu-Jie Hu, I-Ching Ho, Yang-Ming Cheng, Te-Chang Lee

**Affiliations:** 1 Institute of Biomedical Sciences, Academia Sinica, Taipei, Taiwan, Republic of China; 2 Institute of Biopharmaceutical Sciences, National Yang Ming University, Taipei, Taiwan, Republic of China

**Keywords:** cDNA microarray, immortalized urothelial cells, real-time polymerase chain reaction, toxicogenomics, trivalent arsenite, trivalent methylated arsenic compounds

## Abstract

Inorganic arsenic is an environmental carcinogen. The generation of toxic trivalent methylated metabolites complicates the study of arsenic-mediated carcinogenesis. This study systematically evaluated the effect of chronic treatment with sodium arsenite (iAs^III^), monomethylarsonous acid (MMA^III^), and dimethylarsinous acid (DMA^III^) on immortalized human uroepithelial cells (SV-HUC-1 cells) using cDNA microarray. After exposure for 25 passages to iAs^III^ (0.5 μM), MMA^III^ (0.05, 0.1, or 0.2 μM), or DMA^III^ (0.2 or 0.5 μM), significant compound-specific morphologic changes were observed. A set of 114 genes (5.7% of the examined genes) was differentially expressed in one or more sets of arsenical-treated cells compared with untreated controls. Expression analysis showed that exposure of cells to DMA^III^ resulted in a gene profile different from that in cells exposed to iAs^III^ or MMA^III^, and that the iAs^III^-induced gene profile was closest to that in the tumorigenic HUC-1–derived 3-methylcholanthrene–induced tumorigenic cell line MC-SV-HUC T2, which was derived from SV-HUC-1 cells by methylcholanthrene treatment. Of the genes affected by all three arsenicals, only one, that coding for interleukin-1 receptor, type II, showed enhanced expression, a finding confirmed by the reduced increase in NF-κB (nuclear factor kappa B) activity seen in response to interleukin-1β in iAs^III^-exposed cells. The expression of 11 genes was suppressed by all three arsenicals. 5-Aza-deoxycytidine partially restored the transcription of several suppressed genes, showing that epigenetic DNA methylation was probably involved in arsenical-induced gene repression. Our data demonstrate that chronic exposure to iAs^III^, MMA^III^, or DMA^III^ has different epigenetic effects on urothelial cells and represses NF-κB activity.

Arsenic intoxication is a worldwide problem. Billions of people in India, Bangladesh, Inner Mongolia, Taiwan, and North and South America have drunk arsenic-contaminated water for many years (National Research Council 1999) and suffer from a variety of arsenic-induced diseases, such as cancer, diabetes, hypertension, and hyperkeratosis (Chen et al. 1992; Haque et al. 2003; Liu et al. 2002). During the past few decades, arsenic carcinogenesis has been extensively studied using a variety of *in vitro* molecular cytogenetic approaches and *in vivo* animal models (Chen et al. 2004; Kitchin 2001; Rossman 2003; Waalkes et al. 2004). The carcinogenesis-associated effects of arsenic involve genotoxic damage such as chromosomal abnormalities and oxidative stress (Basu et al. 2001; Hei and Filipic 2004; Rossman 2003). In addition to genetic alterations, arsenic exposure was shown recently to induce both global hypomethylation (Xie et al. 2004) or specific hypomethylation of the cyclin D1 and estrogen receptor-α genes (Chen et al. 2004) and hypermethylation of the *p53* (tumor suppressor protein p53) gene (Mass and Wang 1997). Epigenetic alterations caused by modification of DNA methylation are therefore considered to play crucial roles in arsenic carcinogenesis (Sutherland and Costa 2003).

Microarray technology, which measures changes in gene expression at the transcriptional level, is a powerful tool for studying global cellular responses to toxicants (Waters and Fostel 2004). Numerous reports have demonstrated that genes showing aberrant expression after exposure to inorganic trivalent arsenic are involved in signal transduction, cell proliferation, oxidative stress responses, and DNA repair in a variety of cell systems (Bae et al. 2003; Chen et al. 2001a; Hamadeh et al. 2002; Rea et al. 2003; Yih et al. 2002; Zheng et al. 2003) as well as in liver tumors in mice (Chen et al. 2004; Liu et al. 2004). We recently examined gene expression profiles in lymphocytes from an arsenic-exposed human population and found that the expression of several inflammatory molecules is increased after prolonged exposure to arsenic (Wu et al. 2003). Furthermore, we and others have shown that long-term exposure to low concentrations of arsenic causes increased neoplastic transformation of a variety of cells (Achanzar et al. 2002; Chien et al. 2004; Mure et al. 2003; Zhao et al. 1997). These studies strongly suggest that chronic exposure to low levels of arsenic results in epigenetic alterations that may promote arsenic-induced neoplastic transformation or tumor development.

Ingested inorganic arsenic compounds are metabolized by oxidative methylation (Kitchin 2001; Styblo et al. 2002; Thomas et al. 2001). Inorganic pentavalent arsenate (iAs^V^), trivalent arsenite (iAs^III^), and the intermediate metabolites of monomethylarsonic acid (MMA^V^), monomethylarsonous acid (MMA^III^), dimethylarsinic acid (DMA^V^), and dimethylarsinous acid (DMA^III^) have been identified in the urine of arsenic-exposed subjects (Mandal et al. 2004; Wang et al. 2004). MMA^V^ and DMA^V^ are nontoxic, but MMA^III^ and DMA^III^ are more toxic than iAs^III^ for a variety of cell lines (Styblo et al. 2000; Thomas et al. 2001; Vega et al. 2001). MMA^III^ and DMA^III^ are genotoxic for human lymphocytes (Kligerman et al. 2003) and Chinese hamster ovary cells (Dopp et al. 2004) and may interfere with DNA repair to a greater extent than MMA^V^ and DMA^V^ (Schwerdtle et al. 2003). The realization that MMA^III^ and DMA^III^ also cause injury has led to a better understanding of arsenic-mediated carcinogenesis, but their roles in arsenic carcinogenesis remain to be clarified.

To explore the effects of long-term exposure of human urothelial cells to iAs^III^ and its toxic trivalent metabolites, MMA^III^ and DMA^III^, we initiated a systematic study of gene expression changes using cDNA microarray in an SV40-immortalized human urethra–derived urothelial cell line, SV-HUC-1 (HUC-1) (Christian et al. 1987). An HUC-1–derived 3-methylcholanthrene–induced tumorigenic cell line, MC-SV-HUC T2 (MC-T2) (Reznikoff et al. 1988) was also included to examine the relationship between the changes caused by arsenic exposure and tumorigenicity. In this report, we show that chronic exposure to trivalent arsenicals induced compound-specific cell morphologic changes. Different gene expression profiles were produced by exposure to these three trivalent arsenicals, and that caused by iAs^III^ most closely resembled the profile seen in MC-T2 cells. A reduction in the increase in NF-κB (nuclear factor kappa B) activity in response to interleukin 1β (IL1B) treatment was seen in arsenical-exposed cells. 5-Aza-deoxycytidine (5-aza-dC) restoration experiments showed that DNA hypermethylation was involved in the changes in gene expression caused by exposure to trivalent arsenicals.

## Materials and Methods

### Cell culture and arsenic compounds.

HUC-1 cells (CRL-9520) and MC-T2 cells (CRL-9519) were purchased from American Type Culture Collection (Manassas, VA, USA) and grown in F-12 nutrient mixture (Ham) medium (Gibco Invitrogen Life Technologies, Carlsbad, CA, USA) supplemented with 10% fetal calf serum (Hyclone Laboratory, Logan, UT, USA), 2 mM l-glutamine, 100 U/mL of penicillin, and 100 μg/mL streptomycin sulfate at 37°C in a humidified atmosphere containing 5% CO_2_. Both lines were shown to be mycoplasma-free by polymerase chain reaction (PCR) analysis. In nude mice, HUC-1 cells are nontumorigenic, whereas MC-T2 cells form tumors. iAs^III^ (sodium arsenite, Art. 6287) was purchased from Merck KGaA (Darmstadt, Germany). MMA^III^ and DMA^III^ were synthesized in our laboratory, and their chemical structure and purity were verified by nuclear magnetic resonance, mass spectrophotometry, and element analysis.

### Cytotoxicity assay and long-term exposure to iAs^III^, MMA^III^, or DMA^III^.

The cytotoxicity of the three trivalent arsenic compounds for HUC-1 cells was determined using the sulforhodamine B (SRB) colorimetric assay (Skehan et al. 1990). Briefly, 1 × 10^4^ cells in 0.1 mL of F12 medium were seeded in each well of a 96-well plate and incubated for 72 hr with various concentrations of iAs^III^, MMA^III^, or DMA^III^. The cells were then fixed with 50% trichloroacetic acid, stained for 30 min at room temperature with 0.4% SRB in 10% acetic acid, and extracted for 5 min at room temperature with unbuffered 10 mM Tris-base solution, and the absorbance of the extract was measured at 540 nm. The growth-inhibiting and cytotoxic effects of the arsenicals were calculated using the formula [(*A*_treated_ − *A*_zero_)/(*A*_control_ − *A*_zero_)] × 100%, where *A*_zero_ is the absorbance at the time of drug addition and *A*_control_ and *A*_treated_ are the absorbance of untreated and treated wells, respectively, at the end of the experiment. IC_50_ (median inhibitory concentration) values were calculated using CalcuSyn software (Biosoft, Cambridge, UK).

To establish arsenic long-term exposed cell lines, we maintained HUC-1 cells in the presence of trivalent arsenicals at doses giving a 90% cell survival rate, namely, 0.5 μM iAs^III^, 0.2 or 0.5 μM DMA^III^, or 0.05, 0.1, or 0.2 μM MMA^III^. The trivalent arsenical-treated and untreated HUC-1 cells were subcultured twice weekly for at least 20–25 passages before being subjected to microarray assay.

### Preparation of total and poly(A)^+^ RNA.

Total cellular RNA was extracted using TRI reagent (Molecular Research Center, Cincinnati, OH, USA) according to the manufacturer’s instructions. Poly(A)^+^ RNA was then isolated using an Oligotex mRNA midi kit (Qiagen, Hilden, Germany).

### cDNA microarray analysis.

The micro-array chips were made in-house from a collection of approximately 2,000 expressed gene sequences, including genes of potential significance in arsenic toxicity (Wu et al. 2003) and genes selected from subtraction libraries established from oral carcinoma, nasopharyngeal carcinoma, colon cancer, and hepatoma (provided by K.W. Chang, National Yang-Ming University); detailed information on these genes is available from the Institute of Biomedical Sciences, Academia Sinica (Taipei, Taiwan; http://www.ibms.sinica.edu.tw/~bmtcl/As-chip-TCL04.xls). Sources of gene information are GenBank (http://www.ncbi.nih.gov/genbank/) and CGAP (Cancer Genome Anatomy Project; http://cgap.nci.nih.gov/Genes). The cDNA microarray hybridization experiments were performed using a colorimetric detection method as described previously (Wu et al. 2003; Yih et al. 2002). Briefly, cDNA targets were prepared from 2 μg of mRNA using Superscript II reverse transcriptase (Gibco Invitrogen Life Technologies) by incorporating biotin-16-2′-deoxyuridine-5′-triphosphate (Roche Diagnostics, Mannheim, Germany). The labeled cDNA targets were hybridized to the membrane array overnight at 65°C, then the arrays were washed and incubated for 60 min at room temperature with streptavidin-conjugated alkaline phosphatase for chromagen development. After extensive washes to remove any unbound conjugate, substrate solution (0.1 M Tris, pH 9.5, 0.1 M NaCl, 5 mM MgCl_2_, 165 μg/mL nitro-tetrazolium blue chloride, and 87 μg/mL bromo-4-chloro-3-indolyl phosphate *p*-toluidine salt) was added to the array, which was then incubated in the dark at room temperature for 15–20 min with occasional shaking. The intensity of the spots on the arrays was measured using a scanner at an appropriate optical resolution. Quantitative results were obtained using GenePix (version 4.0; Axon Instruments, Union, CA, USA), normalized by a linear regression method, then analyzed and clustered using Spotfire (Spotfire Inc., Somerville, MA, USA). For each treatment, the microarray analysis was repeated 4 times.

### Quantitative real-time reverse-transcriptase PCR.

We used total RNA as the template in the synthesis of first-strand cDNA using an oligo(dT) primer and the AMV reverse transcriptase system (Roche Diagnostics). The PCR primers used to detect the genes of interest and the internal control gene, glyceraldehyde-3-phosphate dehydrogenase (*GAPDH*), are listed in Table 1. Quantitative real-time RT-PCR (Q-PCR) was performed using an ABI PRISM 7700 sequence detection system (Applied Biosystems, Foster City, CA, USA). Thermocycling was performed in a final volume of 10 μL containing 4 μL of cDNA sample, 200 nM of each of the primers, and 5 μL of SYBR green PCR master mix (Applied Biosystems). The relative differences in expression level between genes were expressed using cycle time (C_t_) values as follows: the C_t_ value of the gene of interest was first normalized to that for *GAPDH* in the same sample, then the difference between the treatment and control group was calculated and expressed as an increase or decrease in cycle numbers compared with the control.

### Semiquantitative RT-PCR and agarose gel electrophoresis.

Total RNA was isolated and converted into first-strand cDNA as described above. The cDNA for the test gene was amplified by PCR using the Ampli Taq gold system (Applied Biosystems). The PCR conditions were 94°C for 10 min; 35 cycles of 94°C for 30 sec, 55°C for 30 sec, and 72°C for 30 sec; and 72°C for 7 min. The PCR products were analyzed on a 1.5% agarose gel. *GAPDH* RNA levels were analyzed in parallel to ensure that equal amounts of cDNA were loaded for each reaction

### Immunoblotting analysis.

Cells were harvested in ice-cold phosphate-buffered saline, pH 7.4, using a rubber policemen, then lysed by addition of an equal volume of 2× sodium dodecyl sulfate (SDS) lysis buffer (100 mM Tris HCl, pH 6.8, 200 mM β-mercapto-ethanol, 4% SDS, 0.2% bromophenol blue, and 20% glycerol). The protein concentration was determined using a Bio-Rad Protein Assay system (Bio-Rad Laboratory, Hercules, CA, USA) with bovine serum albumin as standard. Equal amounts (20 μg of protein) of each sample were electrophoretically separated on a 10% SDS polyacrylamide gel and electro-transferred to a PVDF (polyvinylidene difluoride) membrane. After blocking with 4% skim milk, the membranes were probed with primary mouse antibodies against heparin-binding epidermal growth factor-like growth factor (AF-259-NA; R&D System, Inc., Minneapolis, MN, USA), E-cadherin (sc-8426) or keratin 10/13 (sc-6258) (both from Santa Cruz Biotechnology, Inc., Santa Cruz, CA, USA), or integrin β3 (611141; BD Transduction Laboratories, Franklin Lakes, NJ, USA) or goat antibody against β-actin (sc-1616; Santa Cruz Biotechnology, Inc.), followed by secondary horseradish peroxidase–conjugated antibodies against goat or mouse IgG (AP106P or AP124P; Chemicon International Inc., Temecula, CA, USA). Bound antibody was visualized using enhanced SuperSignal West Pico chemiluminescent substrate (Pierce Biotechnology, Inc., Rockford, IL, USA). β-Actin was used as the loading control.

### Transfection and NF-κB activity assay.

To measure NF-κB activity, we used the pNF-κB-Luc reporter (Stratagene, La Jolla, CA, USA) containing five NF-κB enhancer motifs and the coding sequence for firefly luciferase. The pRL-TK plasmid containing *Renilla* luciferase driven by thymidine kinase promoter (Promega Corp., Madison, WI, USA) was used as the internal control. Cells were seeded at 1.5 × 10^5^ per well in 24-well plates the day before and transfected with 0.25 μg of pNF-κB-Luc reporter and 0.05 μg pRL-TK with 0.9 μL FuGene 6 reagent (Roche Diagnostics) following the manufacturer’s instructions. The cells were incubated in the presence of serum-containing medium for 16 hr followed by serum starvation for 24 hr. Human recombinant IL1B (201-LB, R&D System, Inc.) was added as indicated concentrations, and incubation continued for 5 hr. The cells were then harvested, and firefly and *Renilla* luciferase activities were measured using the Dual-Luciferase assay system (E1910, Promega Corp.) according to the manufacturer’s protocol. The NF-κB activity associated with the firefly activity was normalized to the *Renilla* luciferase activity.

### 5-Aza-dC treatment.

HUC-1 cells exposed to 0.2 μM MMA^III^ for 25 passages were incubated for 72 hr with 0, 0.5, or 2.0 μM 5-aza-dC (A3656, Sigma-Aldrich Co.). Passage-matched HUC-1 cells not treated with MMA^III^ or 5-aza-dC were used as controls. The expression level of the test gene in the control HUC-1 cells was taken as 100%.

## Results

### Morphologic changes induced in HUC-1 cells by long-term exposure to iAs^III^, MMA^III^, or DMA^III^.

**Figure 1:**
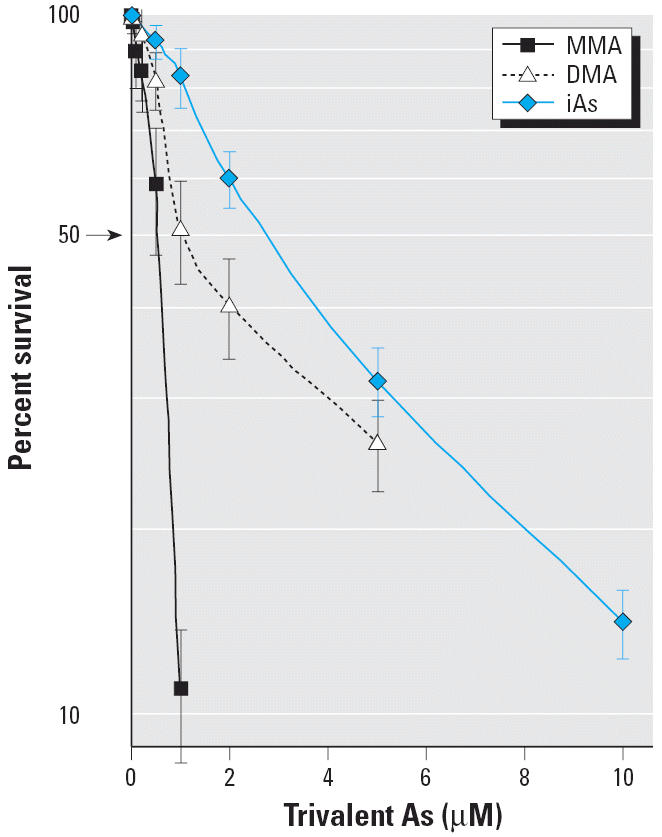
Cytotoxicity of iAs^III^, MMA^III^, and DMA^III^ for HUC-1 cells. HUC-1 cells (1 × 10^4^ cells/well) were seeded in a 96-well plate and exposed for 3 days to various concentrations of iAs^III^, MMA^III^, or DMA^III^; surviving cells were then estimated using the SRB colorimetric assay. The data are the mean ± SD for at least three independent experiments.

To establish an *in vitro* culture system for studying the chronic effect of arsenicals on urothelial cells, we determined the toxicity of three trivalent arsenicals iAs^III^, MMA^III^, and DMA^III^ for nontumorigenic HUC-1 cells using the SRB colorimetric assay (Figure 1) and found the IC_50_ values to be 2.91 ± 0.58, 0.46 ± 0.11, and 1.59 ± 0.26 μM, respectively. For long-term treatment, HUC-1 cells were continuously grown in the presence of trivalent arsenicals at a dose lower than that giving 90% cell survival, that is, 0.05, 0.1, or 0.2 μM MMA^III^; 0.2 or 0.5 μM DMA^III^; and 0.5 μM iAs^III^. Control HUC-1 cells were processed in parallel without arsenicals.

**Figure 2:**
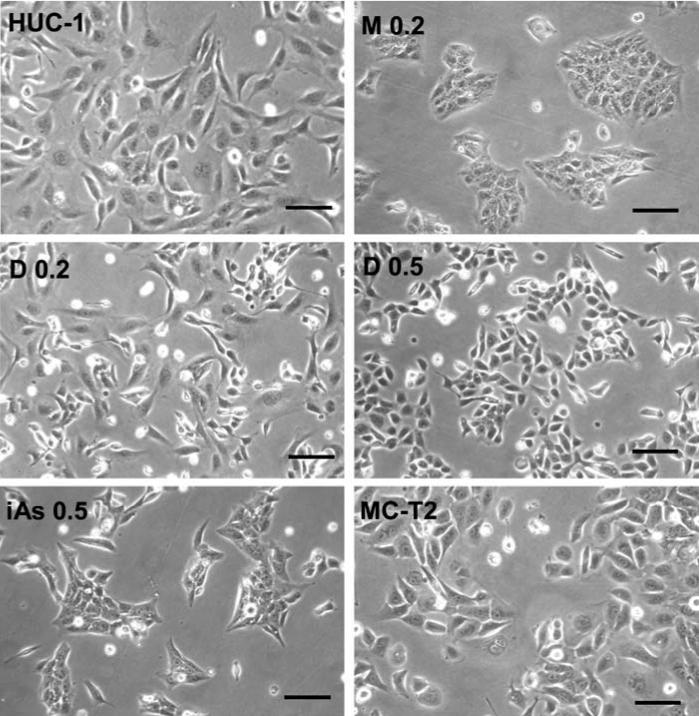
Photomicrographs of HUC-1 cells after long-term exposure to different arsenicals. The morphology of HUC-1 cells left untreated or incubated with 0.2 μM MMA^III^ (M 0.2), 0.2 or 0.5 μM DMA^III^ (D 0.2, D 0.5), or 0.5 μM iAs^III^ (iAs 0.5) for 23–25 passages was examined under a phase-contrast microscope. Tumorigenic MC-T2 cells were included as controls. Bars = 100 μm.

After *in vitro* maintenance for 25 passages, the morphology of the untreated HUC-1 cells was unchanged, but long-term exposure to arsenicals resulted in significant changes. HUC-1 cells exposed to iAs^III^ or MMA^III^ tended to aggregate, were smaller in size than untreated cells (Figure 2), and required longer trypsin digestion to detach them from the surface (data not shown), indicating increased adhesion activity. Long-term exposure to DMA^III^ induced morphologic changes in a concentration-dependent manner, and these differed from those induced by iAs^III^ and MMA^II^; DMA^III^ did not induce significant cell aggregation, but the size of the cells was reduced by exposure to 0.2 μM DMA^III^, and this effect was greater using 0.5 μM DMA^III^. These results show that chronic exposure to trivalent arsenic compounds induced differential morphologic changes in urothelial cells and that none of the induced morphologies resembled that of tumorigenic MC-T2 cells. The proliferation activity of cells cultured in the presence of each arsenical was 70–80% that of untreated controls except for cells exposed to 0.5 μM DMA^III^ for which the proliferation rate was 40–50% that of untreated controls. The tumorigenicity assay showed that HUC-1 cells exposed long term to these trivalent arsenicals were still nontumorigenic in nude mice after subcutaneous injection of 5 × 10^6^ cells, which is in contrast to the results of our previous study (Chien et al. 2004) showing that HaCaT cells (immortalized but non-tumorigenic human keratinocytes) become tumorigenic in nude mice after long-term exposure to low-dose iAs^III^.

### Induction of different gene profiles in HUC-1 cells after long-term exposure to iAs^III^, MMA^III^, or DMA^III^.

**Figure 3:**
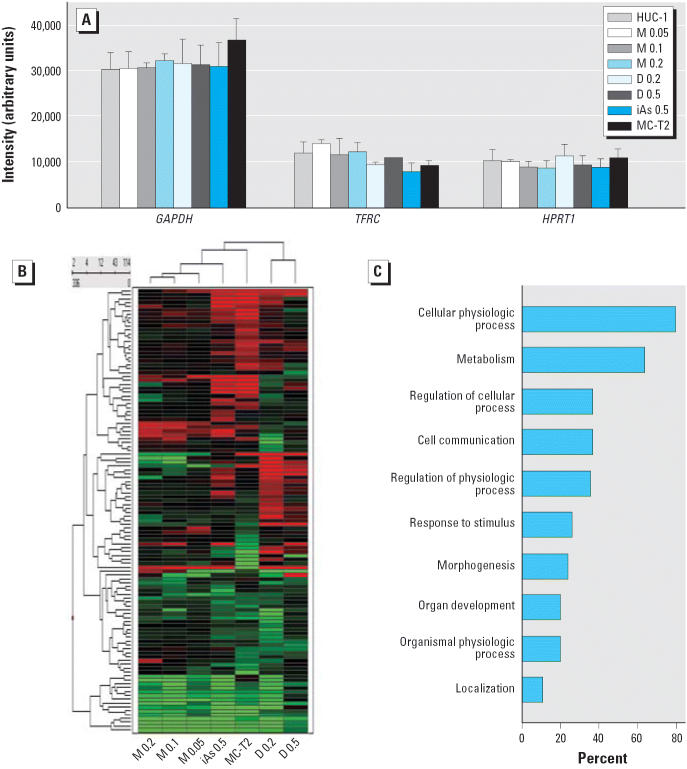
Analysis of changes in gene expression induced by trivalent arsenicals. Abbreviations: D, DMA^III^; M, MMA^III^. (*A*) Intraslide expression levels for the internal control genes, glyceraldehyde-3-phosphate dehydrogenase (*GAPDH*), transferrin receptor (*TFRC*), and hypoxanthine phosphoribosyltransferase 1 (*HPRT1*). (*B*) Hierarchical dendrogram of the gene expression changes. A set of 114 genes showing differential expression in one or more of the arsenical-treated cell lines or MC-T2 cells compared with untreated HUC-1 cells was subjected to hierarchical clustering using the Spotfire clustering program. Increased mRNA levels are shown as shades of red, and decreased levels as shades of green. Names of genes are listed in Supplementary Material, Table 1 (http://ehp.niehs.nih.gov/members/2005/8174/suppl.pdf). (*C*) Gene Ontology-based analysis.

To evaluate global changes in gene expression induced by long-term exposure to low-dose iAs^III^, MMA^III^, or DMA^III^, we used a colorimetric cDNA array (Wu et al. 2003; Yih et al. 2002). We used passage-matched untreated HUC-1 cells as the negative control, and included tumorigenic MC-T2 cells to examine the relationship between changes induced by arsenic exposure and tumorigenesis. As shown in Figure 3A, the intensity (arbitrary units) of the signal for the internal control gene *GAPDH* was similar in untreated and arsenical-treated cells (*p* = 0.3008 by analysis of variance), and similar results were obtained for two other commonly used human housekeeping genes, the transferrin receptor (p90, CD71) (*TFRC*) and hypoxanthine phosphoribosyltransferase 1 (*HPRT1*), except that *TFRC* levels in iAs^III^-treated HUC-1 cells were slightly, but significantly, lower than those in untreated HUC-1 cells. These results showed that the intraassay variation was acceptable.

We selected a set of 114 differentially expressed genes (5.7% of the tested genes) (Supplementary Material, Table 1; http://ehp.niehs.nih.gov/members/2005/8174/suppl.pdf) on the basis of the criteria that there was a significant difference (*p* < 0.05, Student’s *t*-test, *n* = 4) between the expression of the gene in untreated HUC-1 cells and in treated HUC-1 cells and/or MC-T2 cells, and that the ratio for the expression in one or more sets of treated HUC-1 cells and/or MC-T2 cells compared with the expression in untreated HUC-1 cells was > 1.9 or < 0.25. A hierarchical dendrogram of the 114 selected genes is shown in Figure 3B. The expression profiles in cells exposed to different concentrations of MMA^III^ or DMA^III^ were clearly clustered as separate groups. The gene expression pattern most closely resembling that in MC-T2 cells was seen in the iAs^III^-exposed HUC-1 cells. The profile of down-regulated genes in HUC-1 cells exposed to MMA^III^, especially at 0.2 μM, also shared similarities with the MC-T2 cell profile. DMA^III^-treated HUC-1 cells showed a profile distinct from those in the other cells. Together, these gene expression profiles show that these three different trivalent arsenic compounds had different effects on the cells.

To characterize the trivalent arsenical-responsive genes, the selected 114 genes were subjected to Gene Ontology–based biologic property analysis (FatiGO; http://www.fatigo.org/) (Al-Shahrour et al. 2004). Ninety-eight genes of known annotation were submitted, of which 85 were acceptable for level 3 biologic process analysis (Figure 3C). The major gene population (80%) was categorized as “cellular physiologic processes” covering the processes of cell growth and maintenance, cell death, mobility, and transport. In addition, 61.8% of the genes were associated with metabolism (metabolism of nucleic acids, proteins, fatty acids, and vitamin B_6_; protein phosphorylation and targeting; RNA processing; ubiquitin-dependent proteolysis; and regulation of transcription), 36.5% with cell communication (cell adhesion, cell–cell signaling, and signal transduction), 25.9% with responses to stimuli (endogenous and exogenous), and 23.5% with morphogenesis (cellular morphogenesis and organogenesis).

### Confirmation of gene expression profiles by Q-PCR and immunoblotting assays.

**Figure 4:**
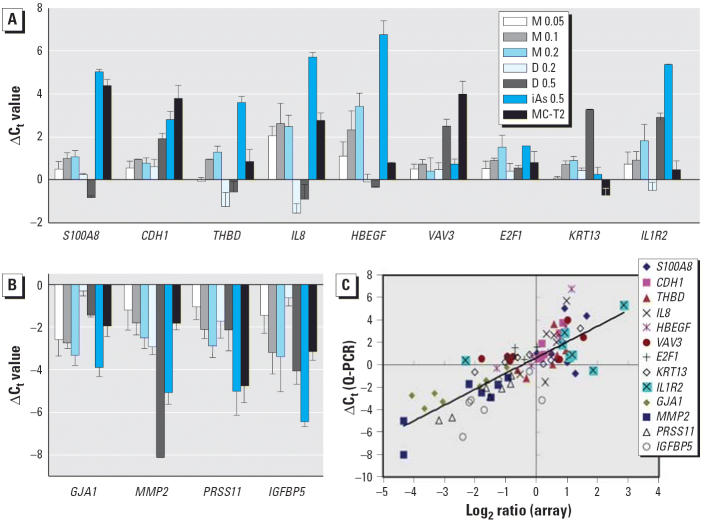
Confirmation of the microarray results by Q-PCR analysis of gene transcripts: increased (*A*) or decreased (*B*) levels both in HUC-1 cells exposed to arsenicals and in MC-T2 cells. Total RNA isolated from iAs^III^-, MMA^III^-, or DMA^III^-treated HUC-1 cells or MC-T2 cells was used in the Q-PCR assay as described in “Materials and Methods.” Error bars represent the SD for triplicate samples from one experiment. (*C*) Semilog scatterplot of gene expression levels measured by microarray (log_2_ of ratio) and Q-PCR (ΔC_t_). *y* = 1.4089*x* + 0.6318; *R*^2^ = 0.6396.

The arsenical-induced changes in gene expression identified by microarray analysis (Supplementary Material, Table 1; http://ehp.niehs.nih.gov/members/2005/8174/suppl.pdf) were checked by Q-PCR. Thirteen of the 114 genes were tested. These 13 included 2 genes [S100 calcium binding protein A8 (*S100A8*) and E-cadherin (*CDH1*)] showing no change or enhanced expression in all sets of arsenical-treated HUC-1 cells in the microarray assay, 4 genes [connexin 43 (*GJA1*), matrix metalloproteinase type 2 (*MMP2*), protease, serine 11 (IGF binding) (*PRSS11*), and insulin-like growth factor binding protein 5 (*IGFBP5*)] showing decreased expression in all sets of arsenical-treated HUC-1 cells, and 7 genes [thrombomodulin (*THBD*), interleukin-8 (*IL8*), heparin-binding epidermal growth factor-like growth factor (*HBEGF*), Vav 3 oncogene (*VAV3*), E2F transcription factor 1 (*E2F1*), keratin 13 (*KRT13*), and interleukin-1 receptor, type II (*IL1R2*)] showing up- or down-regulation of expression in some, but not all, sets of arsenical-treated HUC-1 cells. Figure 4A and B shows the transcriptional differences between untreated HUC-1 cells and arsenical-treated HUC-1 cells or MC-T2 cells measured by Q-PCR assay. To examine the correlation between gene expression changes measured by microarray and by Q-PCR, we analyzed 91 pairs of data for 13 genes in HUC-1 cells exposed to the different concentrations of the three arsenicals and in MC-T2 cells. As shown in Figure 4C, in most cases, gene expression levels measured by the two methods showed a positive correlation, being either up- or down-regulated in both cases (quadrants I and III). Inconsistent results were seen in quadrant II for *VAV3* in 0.05 μM MMA^III^-exposed cells, *KRT13* in 0.1 μM MMA^III^-exposed cells, *E2F1* in 0.1 and 0.2 μM MMA^III^-exposed cells, and *IL1R2* in 0.2 μM DMA^III^-exposed cells; and in quadrant IV *S100A8* in 0.5 μM DMA^III^-exposed cells, *IL8* in 0.2 μM DMA^III^-exposed cells, and *IL1R2* and *IGFBP5* in MC-T2 cells.

**Figure 5:**
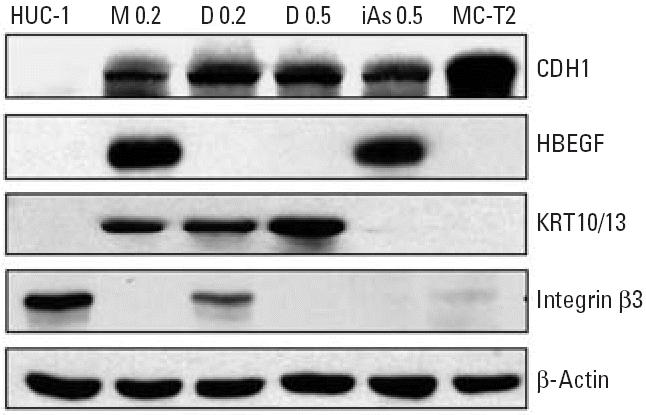
Western blot analysis of arsenical-treated HUC-1 cells and MC-T2 cells. Cell extracts from untreated passage-matched HUC-1 cells, arsenical-treated HUC-1 cells, and MC-T2 cells were separated by SDS–PAGE and immunoblotted with the indicated antibodies as described in “Materials and Methods.”

The results of immunoblotting analysis confirmed the changes in gene expression at the protein level because protein levels of CDH1, HBEGF, and KRT13 in arsenical-treated cells and MC-T2 cells (Figure 5) agreed well with the mRNA levels measured by Q-PCR assay (Figure 4A). In addition, protein expression of integrin β3 (*ITGB3*) (Figure 5) was consistent with transcript levels determined by microarray.

### Distinct gene alterations in HUC-1 cells after long-term exposure to iAs^III^, MMA^III^, or DMA^III^.

To determine which genes were affected similarly by these three arsenicals and which responded to specific arsenic compounds, for further comparative analysis, we selected those genes showing at least a 1.5-fold higher or 2-fold lower expression in HUC-1 cells exposed to 0.5 μM iAs^III^, 0.2 μM MMA^III^, or 0.5 μM DMA^III^ compared with untreated HUC-1 cells.

**Figure 6:**
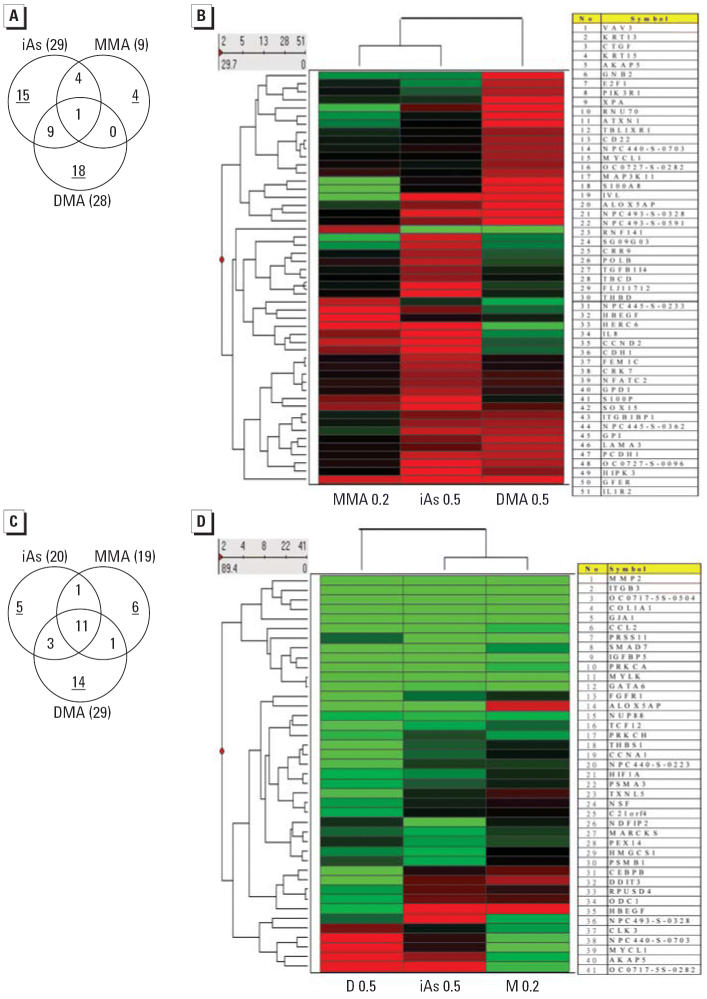
Analysis of altered gene expression in cells exposed to different trivalent arsenicals. (*A, C*) Venn diagram analysis of overlapping sets of iAs^III^-, MMA^III^-, or DMA^III^-enhanced (*A*) or -suppressed (*C*) genes. Numbers in the overlapping regions represent genes modulated in the same direction by two or three trivalent arsenicals. The number of genes modulated uniquely by a single arsenical is underlined. Numbers in parentheses indicate the number of gene modulated after exposure to each arsenic compound. (*B*, *D*) Hierarchical dendrogram analysis of the enhanced (*B*) and suppressed (*D*) genes.

Enhanced expression of 29, 9, and 28 genes was seen, respectively, in iAs^III^-, MMA^III^-, and DMA^III^-exposed cells (Table 2). Of these, *IL1R2* was the only gene showing enhanced expression in response to all three trivalent arsenic compounds (Figure 6A). Of the genes showing enhanced expression in iAs^III^-, MMA^III^-, or DMA^III^-exposed cells, the expression of 15 of 29 (51.7%), 4 of 9 (44.4%), and 18 of 28 (64.3%), respectively, was enhanced uniquely by this one agent (Figure 6A). A hierarchical dendrogram of all 51 genes affected by one or other of the arsenicals is shown in Figure 6B. Clearly, these three trivalent arsenicals induced the expression of distinct patterns of genes.

In addition, suppressed expression was seen for 20, 19, and 29 genes in iAs^III^-, MMA^III^-, and DMA^III^-exposed cells, respectively (Table 3). The number of genes uniquely suppressed in iAs^III^-, MMA^III^-, or DMA^III^-exposed cells was 5 (25%), 6 (31.6%), and 14 (48.3%), respectively (Figure 6C). The higher percentage of genes uniquely suppressed in DMA^III^-exposed cells shows that exposure to DMA^III^ may confer a more diverse gene pattern than exposure to the other two arsenic compounds, consistent with the hierarchical cluster analysis results (Figure 3B). However, in contrast to the single gene showing enhanced expression on exposure to all three agents, 11 genes showed suppressed expression on exposure to all three agents (Figure 6C). Ten are of known function [*GJA1*, *ITGB3*, collagen type I alpha 1 (*COL1A1*), *IGFBP5*, *MMP2*, myosin light polypeptide kinase (*MYLK*), GATA binding protein 6 (*GATA6*), chemokine (C-C motif) ligand 2 (*CCL2*), protein kinase C alpha (*PRKCA*), and nucleoporin (*NUP88*)], and one has unknown function. The hierarchical dendrogram for this set of 41 genes (Figure 6D) showed that the genes suppressed by each of these three trivalent arsenicals shared greater similarity than the enhanced genes (Figure 6B).

### Repression of the IL1B-induced increase in NF-κB activity in iAs^III^-exposed HUC-1 cells.

**Figure 7:**
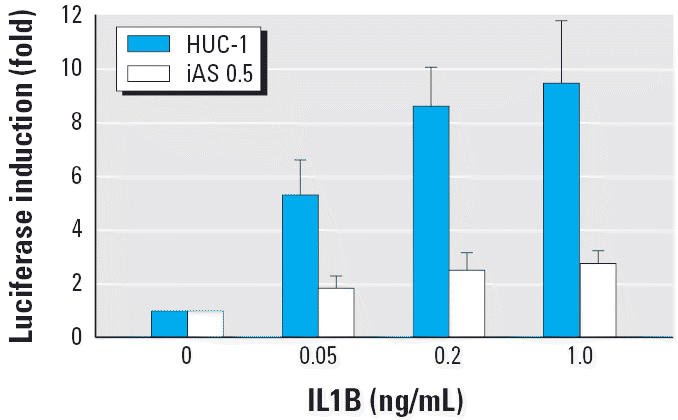
Decreased IL1B-induced NF-κB activity in chronic iAs^III^-exposed HUC-1 cells. HUC-1 cells exposed to 0.5 μM iAs^III^ for 25 passages and passage-matched untreated HUC-1 cells were transfected with NF-κB reporter and control plasmids and then treated with 0, 0.05, 0.2, or 1.0 μg/mL recombinant human IL1B. At the end of treatment, firefly and *Renilla* luciferase activities were determined as described in “Materials and Methods.” Triplicate samples were assayed in each experiment. Data are the mean ± SD for three independent experiments.

Venn diagram analysis showed that *IL1R2* was the only gene to show enhanced expression after exposure to the three test arsenicals. IL1R2 is one of the receptors of inflammatory cytokine IL1; however, because of the lack of a cytoplasmic signaling domain, binding to IL1R2 attenuates IL1-mediated signal transduction, such as the IL1B-induced increase in NF-κB activation (Re et al. 1996). To investigate the effect of overexpression of *IL1R2* on NF-κB signaling, we examined the transgenic NF-κB–dependent promoter activity of arsenical-exposed cells in response to IL1 stimulation. As shown in Figure 7, in untreated HUC-1 cells, the activity of the NF-κB–dependent promoter was increased by treatment with IL1B, and the effect was dose-dependent, with increases of 5.3-fold at 0.05 ng/mL and 8.6-fold at 0.2 ng/mL, then plateauing. In contrast, in cells exposed long-term to iAs^III^, the transgenic NF-κB activity showed only a slight increase in response to IL1B. These results demonstrate that the IL1-mediated NF-κB signaling cascade was impaired in HUC-1 cells exposed long term to iAs^III^.

### A methyltransferase inhibitor reactivates arsenic-suppressed genes.

**Figure 8:**
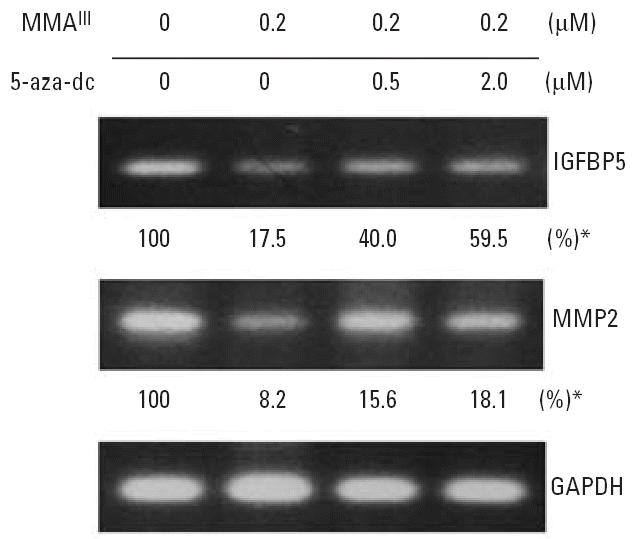
Semi-quantitative analysis of transcript levels in 5-aza-dC–treated cells. HUC-1 cells maintained in the presence of 0.2 μM MMA^III^ for 25 passages were left untreated or treated with 5-aza-dC for 3 days as described in “Materials and Methods,” and then RT-PCR (gels) and Q-PCR (percentages) were used to analyze *IGFBP5* and *MMP2* mRNA levels in these cells and control cells. *Mean of triplicate samples from one Q-PCR analysis.

Gene expression can be inactivated through a wide spectrum of mechanisms. To determine whether DNA hypermethylation silencing was involved in arsenic-induced gene suppression, we tested a methyltransferase inhibitor, 5-aza-dC, for its ability to restore *IGFBP5* and *MMP2* mRNA levels (measured by semiquantitative RT-PCR and Q-PCR) in HUC-1 cells treated with arsenicals. As shown in Figure 8, chronic exposure to 0.2 μM MMA^III^ significantly reduced the expression of *IGFBP5* and *MMP2* to 17.5 and 8.2% of control levels, respectively. However, after 72 hr treatment with 0.5 or 2.0 μM 5-aza-dC, the expression of both genes increased. Using 2 μM 5-aza-dC, *IGFBP5,* and *MMP2* gene expression increased 3.4- and 2.2-fold, respectively (Figure 8). These results suggest that at least part of the MMA^III^-induced repression of genes is dependent on DNA methylation.

## Discussion

Toxicogenomics is a powerful tool for studying the interaction between genes and environmental stress in disease causation (Waters and Fostel 2004). The results of a microarray-based study performed to give a comprehensive idea of the effects of trivalent arsenic compounds on human urothelial cells showed that long-term exposure of HUC-1 cells to nonlethal doses of iAs^III^, MMA^III^, or DMA^III^ had distinct effects on a variety of cellular responses. On the basis of the morphology (Figure 2), the dendrogram (Figure 3A), and the gene profile analyses (Figure 6), the DMA^III^-induced cellular and molecular changes differed from those induced by iAs^III^ or MMA^III^. In addition, the gene expression pattern induced by iAs^III^ most closely resembled that in tumorigenic MC-T2 cells. However, each of these three compounds can cause DNA damage and cytogenetic alterations in mammalian cells (Dopp et al. 2004; Kligerman et al. 2003), inhibit nucleotide excision repair (Schwerdtle et al. 2003), and induce morphologic transformation in Syrian hamster embryo cells (Lee et al. 1985; Ochi et al. 2004). The effects on cancer development of the distinct changes in gene expression caused by low-dose long-term exposure to these three trivalent arsenic compounds require further clarification.

It is important to note that the HUC-1 cells used in the present study were derived by SV40 transformation (Christian et al. 1987). The transforming ability of SV40 is mediated by a large T-antigen oncoprotein binding to, and inactivating, gatekeeper tumor suppressor proteins, such as p53 and retinoblastoma (RB) (Pipas and Levine 2001). p53 protein is a crucial mediator of cellular responses to DNA damage and can initiate a program of cell-cycle arrest, cellular senescence, or apoptosis (Harris and Levine 2005). In the absence of functional p53 protein, cells lose genomic integrity and are prone to cancer development. p53 functions as a transcription factor up-regulating many target genes, such as *p21* (cyclin-dependent kinase inhibitor 1A, Cip1), *MDM2* (transformed 3T3 cell double minute 2), and *GADD45* (growth arrest and DNA-damage-inducible, alpha) (Levine 1997). A recent report (Liu et al. 2003) has shown that myeloma cells lacking p53 are more sensitive to arsenic trioxide (ATO), which induces apoptosis and arrest of the cells in G2/M phase of the cell cycle, whereas cells with wild-type p53 are relatively resistant to ATO and are arrested in G_1_ phase by ATO. Thus, cells containing a wild-type or mutated/null p53 may show different cellular responses to arsenicals. Our findings showing transcriptional alterations mediated by chronic exposure to arsenic species in cells lacking functional p53 protein might therefore differ from those seen in cells with functional p53.

Our microarray profiling showed that several of the genes responding to chronic exposure to all three arsenicals were involved in the regulation of signal transduction pathways. Of the significantly affected genes in the total of 2,000 tested, that coding for *IL1R2* was the only one to show enhanced expression after long-term exposure to each of the three trivalent arsenicals. IL1R2 is a nonsignaling decoy receptor of the strong proinflammatory cytokine IL1. Binding of IL1 to IL1R2 attenuates IL1-induced inflammatory responses, such as NF-κB activation (Dower 2000; Wald et al. 2003). Our results (Figure 7) showed that the increased *IL1R2* expression caused by the trivalent arsenicals disrupted signal transduction mediated by IL1B. Alterations of cellular signal transduction are suggested to play crucial roles in arsenic-mediated carcinogenesis (Qian et al. 2003). Arsenic has been shown to activate the stress-induced kinase pathway of MAPK p38, ERK (extracellular signal-regulated kinase), and JNK (c-Jun N-terminal kinase) (Cavigelli et al. 1996; Liu et al. 1996) and to repress the STAT (signal transducers and activators of transcription) signaling pathway (Hayashi et al. 2002). It can also either stimulate or inhibit NF-κB activation depending on the concentration, exposure time, and cell type (Harris and Shi 2003). For instance, arsenite or ATO has been shown to induce apoptosis of melanoma and lymphoma cells by inhibiting NF-κB activity (Ivanov and Hei 2004; Mathas et al. 2003); however, ATO enhances CD95/Fas-induced apoptosis through NF-κB activation (Woo et al. 2004). Further studies are required to characterize the biologic significance of the overexpressed IL1R2 and suppression of NF-κB activity in urothelial cells chronically exposed to arsenic.

Unexpectedly, the expression of genes that are negatively associated with carcinogenesis was also enhanced by arsenical exposure. For instance, expression of *CDH1*, a major component involved in cell–cell adhesion and frequently negatively associated with metastatic and invasive carcinomas (Thiery 2002), was enhanced in iAs^III^-exposed cells, and expression of its precursor protein, protocadherin 1, was enhanced in DMA^III^-exposed cells. THBD is a cell-surface–expressed glycoprotein playing a role in anticoagulation (Van de Wouwer et al. 2004), and higher *THBD* expression in tumors correlates with better survival and reduced metastatic activity (Weiler and Isermann 2003). *THBD* expression was enhanced in MMA^III^- or iAs^III^-exposed HUC-1 cells. The present results suggest that a wide spectrum of cellular responses are induced to cope with chronic nonlethal toxic stresses.

In contrast to the single gene activated by all three trivalent arsenicals, 11 genes were suppressed by all three. Several of these are also involved in signal transduction. For instance, integrins, heterodimeric transmembrane receptors composed of an α-chain and a β-chain, are the major component of the focal adhesive complex that regulates stress-fiber formation, cell shape migration, and signal transduction via molecules such as phosphatidylinositol 3-kinase and NF-κB (Filardo et al. 1995; Guo and Giancotti 2004; Hood and Cheresh 2002). A recent report has shown that sodium arsenite reduces focal adhesions and the phosphorylation of focal adhesion kinase and paxillin (Yancy et al. 2005), supporting the idea that focal adhesions and the downstream signal transduction pathways may be targets of chronic arsenical exposure. GJA1 is a gap-junction channel protein involved in cell communication (Aasen et al. 2003). A recent study has revealed the novel paradigm that GJA1 acts as a positive regulator of the NF-κB signaling cascade and that GJA1 not only is crucial in cell communication but also plays a role in signal transduction regulation (Matsuda et al. 2003). MYLK is one of the cytoskeleton kinases involved in focal adhesion and is also associated with NF-κB activity induced by tumor necrosis factor-α (Wadgaonkar et al. 2005). Taken together, our results suggest that many signal transduction cascades may be modulated by chronic exposure to trivalent arsenicals. Imbalanced expression of genes in response to stress has been suggested to be involved in carcinogenesis (Hofseth 2004). The present study revealed that arsenic induced toxicopathologic injuries and that cellular adaptation to arsenic was associated with alterations in the expression of a complex of genes.

Generation of oxidative stress has been proposed to be involved in the toxicity induced by low-dose arsenic (Schoen et al. 2004). The expression of many oxidative stress–responsive genes is induced by arsenic exposure; examples include superoxide dismutase 1 and NAD(P)H quinone oxidoreductase, which respond to oxidative stress generated by arsenic metabolic methylation (Hamadeh et al. 2002); heme oxygenase 1, a marker for arsenic-induced stress (Liu et al. 2001b); and glutathione (Brambila et al. 2002), heat-shock protein (Barnes et al. 2002), and multidrug-resistant efflux transporters (Liu et al. 2001a), which are induced during the acquisition of tolerance to arsenic. These genes showed no major changes in our gene profiles, although they were included on the array. The differences in expression profiles obtained in our system and in others may be due to tissue-specific variation but are more likely due to different dosages and exposure times. In our system, we applied iAs^III^, MMA^III^, and DMA^III^ at doses of 0.05 to 0.5 μM for more than 3 months (~ 25 passages), whereas the other studies mentioned above involved the use of 0.05–25 μM iAs^III^ for periods of only several hours to 2 weeks. Our results therefore suggest that these oxidative stress–related genes may be involved in the carcinogenic process at a period earlier than the window of this study.

Toxicant-induced gene expression changes and cancer have been shown to be associated with epigenetic modifications (Sutherland and Costa 2003). Methylation of CpG islands appears to be responsible for the transcriptional silencing of critical genes in various types of cancer (Egger et al. 2004; Jones and Baylin 2002). Exposure of cells to arsenic results in global or specific hypomethylation (Chen et al. 2001b, 2004; Xie et al. 2004) or hypermethylation of specific genes such as tumor suppressor *p53* (Mass and Wang 1997). Our microarray analysis showed that the number of genes suppressed by all three agents was much higher than the number activated by all three, suggesting that down-regulation of genes might be an effect common to all three of these trivalent arsenicals. Furthermore, the methyltransferase inhibitor 5-aza-dC partially restored transcription of IGFBP5 and MMP2, again implying that epigenetic gene regulation may be involved in chronic arsenical–mediated gene modulation.

The various forms of arsenicals to which humans are exposed complicate studies of the mechanism of arsenic-mediated carcinogenesis. In addition, the lack of appropriate animal models and sensitive biomarkers for monitoring the adverse effects of arsenic has also limited progress. However, recent investigations have revealed that arsenic may exert its adverse effects via epigenetic pathways. Because biologic pathways are connected through a complicated network, the ability to identify and confirm the roles of arsenic-induced differentially expressed genes during cancer development would help us understand how a normal cell is transformed into a neoplasm and to search for new intervention regimes. The characterization of the gene expression profiles induced by arsenicals may therefore provide an understanding of their roles in carcinogenesis.

## Supplementary Material

Supplemental Figures and Tables

## Figures and Tables

**Table 1:** Primer sequences used for Q-PCR and RT-PCR.^a^

Gene	Forward primer	Reverse primer
*CDH1*	AACGCATTGCCACATACACTCT	CATTCTGATCGGTTACCGTGATC
*E2F1*	GAGCAGATGGTTATGGTGATCAAA	AGGGCACAGGAAAACATCGA
*GAPDH*	GGAGTCCCTGCCACACTCA	GCCCCTCCCCTCTTCAAG
*GJA1*	CCAAAGACTGTGGGTCTCAA	CTCACTTGCTTGCTTGTTGTA
*HBEGF*	AGGACTTCTGCATCCATGGA	TGGTCATAGGTATATAAGCGAT
*IGFBP5*	CTCACTTGCTTGCTTGTTGTA	GCACTGCTTTCTCTTGTAGAA
*IL1R2*	TGTGCTGGCCCCACTTTC	GCACAGTCAGACCATCTGCTTT
*IL8*	GTGCAGTTTTGCCAAGGA	TTCTGTGTTGGCGCAGTG
*KRT13*	GCGGGACTACAGCCCCTACTAC	CCAGCCTGGCATTGTCAAT
*MMP2*	GCACTGCTTTCTCTTGTAGAA	GATCTGAGCGATGCCATCAA
*PRSS11*	CCGTGGTTCATATCGAATTGT	CCGTGGTTCATATCGAATTGT
*S100A8*	TGGAGAAAGCCTTGAACTCTATCA	GGTCTCTAGCAATTTCTTCAGGTCAT
*THBD*	GAGGACGTGGATGACTGCATACT	GGTACTCGCAGTTGGCTCTGA
*VAV3*	GAACAAGGGACACTCAAACTACCA	GTTCCTAATGACCTGCATCTTTGG

a Primer sequences were designed by Primer Express 2.0 (Applied Biosystems) according to their sequence ID shown in Supplementary Material, Table 1 (http://ehp.niehs.nih.gov/members/2005/8174/suppl.pdf).

**Table 2:** Genes showing enhanced expression in HUC-1 cells exposed long-term to MMA^III^, DMA^III^, or iAs^III^.

Cluster and gene name	Gene symbol	Function
MMA^III^, DMA^III^, and iAs^III^		
Interleukin-1 receptor, type II	*IL1R2*	Interleukin-1, type II, blocking receptor activity
MMA^III^ specific		
Arachidonate 5-lipoxygenase-activating protein	*ALOX5AP*	Leukotriene biosynthesis
Thrombomodulin	*THBD*	Receptor activity
DMA^III^ specific		
A kinase (PRKA) anchor protein 5	*AKAP5*	Signal transduction
Ataxin 1	*ATXN1*	RNA binding
CD22 antigen	*CD22*	Cell adhesion
Connective tissue growth factor	*CTGF*	Regulation of cell growth, cell adhesion
RNA, U70 small nucleolar	*RNU70*	Unknown
Integrin beta 1 binding protein 1	*ITGB1BP1*	Cell adhesion
E2F transcription factor 1	*E2F1*	G_1_ phase of mitotic cell cycle
Guanine nucleotide binding protein (G protein), beta polypeptide 2	*GNB2*	signaling pathway
Keratin 13	*KRT13*	Intermediate filament
Keratin 15	*KRT15*	Intermediate filament
Laminin, alpha 3	*LAMA3*	Cell surface receptor
Phosphoinositide-3-kinase, regulatory subunit, polypeptide 1	*PIK3R1*	Phosphatidylinositol 3-kinase activity
Protocadherin 1	*PCDH1*	Cell–cell signaling
Transducin (beta)-like 1X-linked receptor 1	*TBL1XR1*	Regulation of transcription
Vav 3 oncogene	*VAV3*	Small GTPase-mediated signal transduction
V-myc myelocytomatosis viral oncogene homolog 1	*MYCL1*	Transcription factor activity
Xeroderma pigmentosum, complementation group A	*XPA*	Nucleotide-excision repair
iAs^III^ specific		
E-cadherin	*CDH1*	Cell–cell adhesion
CDC2-related protein kinase 7	*CRK7*	Protein kinase activity
Tubulin-specific chaperone d	*TBCD*	Beta-tubulin folding
Cisplatin resistance related protein CRR9p	*CRR9*	Integral to membrane
Cyclin D2	*CCND2*	Regulation of cell cycle
Polymerase (DNA directed), beta	*POLB*	DNA replication
Ring finger protein 141	*RNF141*	Ubiquitin ligase complex
Nuclear factor of activated T-cells, cytoplasmic, calcineurin-dependent 2	*NFATC2*	Transcription factor activity
S100 calcium binding protein P	*S100P*	Calcium ion binding
Fem-1 homolog c (C. elegans)	*FEM1C*	Unknown
Transforming growth factor beta 1 induced transcript 4	*TGFB1I4*	Transcription factor activity
MMA^III^ and iAs^III^		
Diphtheria toxin receptor (heparin-binding epidermal growth factor-like growth factor)	*HBEGF*	Growth factor activity
DMA^III^ and iAs^III^
Glucose phosphate isomerase	*GPI*	Glucose-6-phosphate isomerase activity, cytokine activity
Growth factor, augmenter of liver regeneration	*GFER*	Cell proliferation
Hect domain and RLD 6	*HERC6*	Ubiquitin cycle
Homeodomain interacting protein kinase 3	*HIPK3*	Protein kinase activity
Interleukin-8	*IL8*	Cytokine activity
Involucrin	*IVL*	Keratinocyte differentiation
SRY (sex determining region Y)-box 15	*SOX15*	Transcription factor activity
Mitogen-activated protein kinase kinase kinase 11	*MAP3K11*	JNK cascade
OC0717-S-0282^a^		
S100 calcium binding protein A8	*S100A8*	Inflammatory response

Gene names and symbols are from UniGene (http://www.ncbi.nlm.nih.gov/entrez/query.fcgi?db=unigene).

a Clone ID of original subtraction libraries.

**Table 3:** Genes suppressed in HUC-1 cells after long-term exposure to MMA^III^, DMA^III^, or iAs^III^.

Cluster and gene name	Gene symbol	Function
MMA^III^, DMA^III^, and iAs^III^		
Chemokine (C-C motif) ligand 2	*CCL2*	Cell–cell signaling
Collagen, type I, alpha 1	*COL1A1*	Extracellular matrix
Connexin 43	*GJA1*	Cell–cell signaling
GATA binding protein 6	*GATA6*	Transcription factor activity
Insulin-like growth factor binding protein 5	*IGFBP5*	Regulation of cell growth
Integrin, beta 3	*ITGB3*	Cell–matrix adhesion
Matrix metalloproteinase 2	*MMP2*	Metallopeptidase activity
Myosin, light polypeptide kinase	*MYLK*	Protein kinase activity
Nucleoporin 88 kDa	*NUP88*	Nuclear pore
Protein kinase C, alpha	*PRKCA*	Cell proliferation
Homo sapiens PAC clone RP5-1057M1 from 7, complete sequence		Unknown
MMA^III^ specific		
A kinase (PRKA) anchor protein 5	*AKAP5*	Signal transduction
CDC-like kinase 3	*CLK3*	Protein kinase activity
OC0717-S-0282^a^		
V-myc myelocytomatosis viral oncogene homolog 1	*MYCL1*	Transcription factor activity
DMA^III^ specific		
CCAAT/enhancer binding protein (C/EBP), beta	*CEBPB*	Transcription factor activity
Cyclin A1	*CCNA1*	Regulation of cell cycle
Heparin-binding epidermal growth factor-like growth factor	*HBEGF*	Growth factor activity
DNA-damage-inducible transcript 3	*DDIT3*	Cell cycle arrest
Fibroblast growth factor receptor 1	*FGFR1*	MAPKKK cascade, protein kinase activity
RNA pseudouridylate synthase domain containing 4	*RPUSD4*	RNA processing
Proteasome (prosome, macropain) subunit, alpha type, 3	*PSMA3*	Ubiquitin-dependent protein catabolism
Hypoxia-inducible factor 1, alpha subunit	*HIF1A*	Response to stress, signal transduction, transcription factor activity
Chromosome 21 open reading frame 4	*C21orf4*	Unknown
*N*-Ethylmaleimide-sensitive factor	*NSF*	ATP-dependent peptidase activity
Ornithine decarboxylase 1	*ODC1*	Catalytic activity
Thioredoxin-like 5	*TXNL5*	Electron transporter activity
Thrombospondin 1	*THBS1*	Cell adhesion, signal transduction, coagulation
iAs^III^ specific		
3-Hydroxy-3-methylglutaryl-coenzyme A synthase 1	*HMGCS1*	Acetyl-CoA metabolism
Myristoylated alanine-rich protein kinase C substrate	*MARCKS*	Actin binding, cell motility
Nedd4 family interacting protein 2	*NDFIP2*	Signal transducer activity
Proteasome subunit, beta type, 1	*PSMB1*	Ubiquitin-dependent protein catabolism
Peroxisomal biogenesis factor 14	*PEX14*	Integral to peroxisomal membrane
MMA^III^ and iAs^III^
Protease, serine, 11 (IGF binding)	*PRSS11*	Serine-type endopeptidase activity, regulation of cell cycle
DMA^III^ and iAs^III^		
Arachidonate 5-lipoxygenase-activating protein	*ALOX5AP*	Leukotriene biosynthesis
SMAD, mothers against DPP homolog 7 (*Drosophila*)	*SMAD7*	Transforming growth factor beta receptor, inhibitory cytoplasmic mediator activity
Transcription factor 12	*TCF12*	Regulation of transcription

Gene names and symbols are from UniGene (http://www.ncbi.nlm.nih.gov/entrez/query.fcgi?db=unigene).

a Clone ID of original subtraction libraries.
