# Supplementary material for: Distinct Gene Expression Profiles in Immortalized Human Urothelial Cells Exposed to Inorganic Arsenite and Its Methylated Trivalent Metabolites
Source: Environ Health Perspect. 2005 Aug 17;114(3):394–403. doi: 10.1289/ehp.8174 (PMC1392234; doi:10.1289/ehp.8174)
Supplement: Supplemental Figures and Tables [file ehp0114-000394s1.pdf]

# Supplementary Material

**Table 1.** List of genes selected for the hierarchical cluster analysis shown in Fig. 3B*.

| No. | Genes** | | | HUC-1 | | | | | | MC-T2 |
|---|---|---|---|---|---|---|---|---|---|---|
| | | | | Arsenicals (μM) | | | | | | |
| | Full name | Symbol | Sequence ID | $MMA^{III}$ | | | $DMA^{III}$ | | $iAs^{III}$ | None |
| | | | | 0.05 | 0.1 | 0.2 | 0.2 | 0.5 | 0.5 | |
| 1 | S100 calcium binding protein A8 | *S100A8* | NM_002964 | 1.60 | 1.41 | 1.01 | 2.02 | 2.41 | 1.90 | 3.13 |
| 2 | OC0727-S-0096[#] | | | 1.05 | 0.92 | 1.12 | 1.94 | 1.68 | 1.76 | 2.82 |
| 3 | Growth factor, augmenter of liver regeneration | *GFER* | NM_005262 | 0.93 | 1.18 | 1.06 | 0.65 | 1.54 | 2.61 | 3.14 |
| 4 | Homeodomain interacting protein kinase 3 | *HIPK3* | NM_005734 | 0.87 | 1.09 | 1.15 | 1.05 | 1.64 | 2.21 | 2.05 |
| 5 | SRY (sex determining region Y)-box 15 | *SOX15* | NM_006942 | 0.77 | 1.05 | 1.58 | 1.14 | 1.34 | 2.02 | 1.59 |
| 6 | Glycerol-3-phosphate dehydrogenase 1 | *GPD1* | NM_005276 | 1.30 | 1.31 | 1.15 | 0.55 | 1.25 | 1.67 | 2.26 |
| 7 | Nuclear factor of activated T-cells, cytoplasmic, calcineurin-dependent 2 | *NFATC2* | NM_012340 | 1.32 | 1.49 | 1.21 | 0.83 | 1.32 | 1.57 | 1.93 |
| 8 | Fem-1 homolog c | *FEM1C* | NM_020177 | 1.25 | 1.20 | 1.16 | 0.82 | 1.14 | 1.66 | 1.84 |
| 9 | CDC2-related protein kinase 7 | *CRK7* | NM_016507 | 1.11 | 1.11 | 1.10 | 0.80 | 1.33 | 1.55 | 1.62 |
| 10 | Lipopolysaccharide-induced TNF factor | *LITAF* | NM_004862 | 1.30 | 1.40 | 1.06 | 0.91 | 1.38 | 1.17 | 1.52 |
| 11 | Kruppel-like factor 5 | *KLF5* | NM_001730 | 0.74 | 0.85 | 0.89 | 1.32 | 0.99 | 1.13 | 1.88 |
| 12 | TAP binding protein | *TAPBP* | NM_003190 | 0.88 | 0.88 | 0.80 | 1.03 | 1.33 | 1.29 | 1.73 |
| 13 | GDP-mannose 4,6-dehydratase | *GMDS* | NM_001500 | 0.87 | 0.90 | 0.74 | 1.02 | 1.21 | 1.06 | 1.51 |
| 14 | Laminin, alpha 3 | *LAMA3* | NM_000227 | 1.03 | 1.01 | 0.98 | 1.37 | 1.78 | 1.49 | 1.83 |
| 15 | NPC440-S-0005[#] | | | 1.02 | 1.29 | 1.07 | 1.48 | 1.23 | 1.23 | 1.93 |
| 16 | NPC440-S-0006[#] | | | 1.01 | 1.18 | 1.10 | 1.52 | 1.33 | 1.23 | 1.62 |
| 17 | Cadherin 1, type 1, E-cadherin | *CDH1* | NM_004360 | 1.04 | 1.18 | 1.12 | 1.12 | 1.14 | 1.70 | 1.82 |
| 18 | Myeloid/lymphoid or mixed-lineage leukemia 3 | *MLL3* | NM_021230 | 0.97 | 1.09 | 1.06 | 1.17 | 1.15 | 1.19 | 1.54 |
| 19 | Ladinin 1 | *LAD1* | NM_005558 | 1.04 | 1.07 | 1.18 | 1.11 | 1.42 | 1.31 | 1.52 |
| 20 | Phosphoinositide-3-kinase, regulatory subunit 1 | *PIK3R1* | NM_181504 | 1.14 | 0.85 | 0.83 | 0.60 | 1.57 | 0.99 | 1.97 |

| | | | | | | | | | | |
|---|---|---|---|---|---|---|---|---|---|---|
| 21 | Transducin (beta)-like 1X-linked receptor 1 | *TBL1XR1* | NM_024665 | 1.07 | 1.07 | 1.10 | 0.67 | 1.57 | 0.93 | 1.70 |
| 22 | OC0727-S-0467# | | | 1.03 | 1.23 | 1.17 | 0.46 | 1.01 | 0.77 | 1.15 |
| 23 | Hect domain and RLD 6 | *HERC6* | NM_017912 | 1.90 | 1.16 | 1.53 | 0.60 | 0.57 | 2.22 | 4.73 |
| 24 | Cyclin D2 | *CCND2* | NM_001759 | 1.30 | 1.64 | 1.50 | 0.81 | 0.67 | 2.82 | 4.22 |
| 25 | NPC493-S-0591# | | | 1.04 | 1.17 | 0.56 | 1.36 | 0.60 | 1.78 | 2.67 |
| 26 | TSC22 domain family 1 | *TGFB1I4* | NM_006022 | 1.10 | 1.02 | 0.94 | 1.57 | 0.75 | 2.56 | 2.86 |
| 27 | Tubulin-specific chaperone d | *TBCD* | NM_005993 | 1.09 | 0.85 | 1.07 | 0.99 | 0.87 | 2.35 | 1.90 |
| 28 | Zinc finger protein 286 | *ZNF286* | NM_020652 | 0.99 | 0.87 | 0.53 | 1.01 | 0.55 | 1.00 | 1.65 |
| 29 | NPC493-S-0328# | | | 0.98 | 0.77 | 0.45 | 0.88 | 0.60 | 1.82 | 1.19 |
| 30 | Ring finger protein 141 | *RNF141* | NM_016422 | 0.97 | 1.01 | 0.93 | 0.71 | 0.69 | 1.60 | 1.68 |
| 31 | Ubiquitin specific protease 21 | *USP21* | NM_012475 | 0.98 | 0.99 | 0.92 | 0.80 | 0.85 | 1.16 | 1.53 |
| 32 | Polymerase (DNA directed), beta | *POLB* | NM_002690 | 1.08 | 1.06 | 1.04 | 0.86 | 0.92 | 1.55 | 1.02 |
| 33 | Cisplatin resistance related protein CRR9p | *CRR9* | NM_030782 | 1.01 | 0.91 | 0.95 | 1.15 | 0.85 | 1.57 | 0.85 |
| 34 | SG09G03# | | | 1.00 | 1.27 | 1.19 | 1.06 | 0.72 | 1.72 | 0.86 |
| 35 | NPC445-S-0233# | | | 1.61 | 1.87 | 2.79 | 0.57 | 0.87 | 1.15 | 1.28 |
| 36 | Thrombomodulin | *THBD* | NM_000361 | 1.45 | 2.13 | 1.95 | 0.80 | 0.65 | 1.49 | 1.60 |
| 37 | Interleukin 8 | *IL8* | NM_000584 | 1.57 | 1.49 | 1.78 | 1.22 | 0.69 | 1.98 | 1.26 |
| 38 | Heparin-binding EGF-like growth factor | *HBEGF* | NM_001945 | 1.39 | 1.71 | 1.95 | 0.89 | 0.41 | 2.23 | 0.92 |
| 39 | DNA-damage-inducible transcript 3 | *DDIT3* | NM_004083 | 1.00 | 1.75 | 1.44 | 1.13 | 0.33 | 1.25 | 1.65 |
| 40 | OC0727-S-0822# | | | 0.74 | 1.34 | 1.25 | 0.66 | 0.27 | 1.05 | 0.94 |
| 41 | CCAAT/enhancer binding protein (C/EBP), beta | *CEBPB* | NM_005194 | 0.90 | 1.13 | 1.19 | 0.82 | 0.45 | 1.08 | 1.05 |
| 42 | Ornithine decarboxylase 1 | *ODC1* | NM_002539 | 1.01 | 1.26 | 1.21 | 0.78 | 0.45 | 1.29 | 0.87 |
| 43 | NPC440-S-0703# | | | 0.44 | 0.36 | 0.36 | 10.73 | 3.16 | 1.12 | 3.54 |
| 44 | V-myc myelocytomatosis viral oncogene homolog 1 | *MYCL1* | NM_005376 | 1.19 | 0.21 | 0.30 | 1.10 | 4.15 | 1.09 | 0.85 |
| 45 | A kinase (PRKA) anchor protein 5 | *AKAP5* | NM_004857 | 0.74 | 0.13 | 0.43 | 1.63 | 1.98 | 1.40 | 0.79 |
| 46 | Vav 3 oncogene | *VAV3* | NM_006113 | 0.29 | 0.52 | 0.55 | 1.70 | 2.91 | 0.59 | 2.03 |
| 47 | Involucrin | *IVL* | NM_005547 | 0.83 | 0.56 | 0.98 | 1.82 | 4.66 | 1.53 | 1.10 |
| 48 | Mitogen-activated protein kinase kinase kinase 11 | *MAP3K11* | NM_002419 | 0.88 | 0.58 | 0.77 | 2.07 | 2.95 | 1.52 | 1.10 |
| 49 | OC0717-S-0282# | | | 0.94 | 0.98 | 0.38 | 1.22 | 2.92 | 2.30 | 1.03 |
| 50 | Protocadherin 1 | *PCDH1* | NM_002587 | 1.15 | 1.00 | 1.11 | 1.50 | 1.69 | 1.39 | 1.09 |
| 51 | NPC445-S-0362# | | | 0.91 | 0.82 | 0.89 | 1.23 | 1.60 | 1.58 | 1.09 |

| | | | | | | | | | | |
|---|---|---|---|---|---|---|---|---|---|---|
| 52 | Xeroderma pigmentosum, complementation group A | *XPA* | NM_000380 | 0.91 | 0.93 | 0.90 | 1.34 | 1.60 | 1.09 | 1.07 |
| 53 | Glucose phosphate isomerase | *GPI* | NM_000175 | 0.79 | 0.75 | 0.76 | 1.30 | 1.66 | 1.77 | 0.73 |
| 54 | Integrin beta 1 binding protein 1 | *ITGB1BP1* | NM_004763 | 0.97 | 1.08 | 0.84 | 0.79 | 1.54 | 1.48 | 0.72 |
| 55 | RNA, U70 small nucleolar | *RNU70* | BF670618 | 1.06 | 0.75 | 0.90 | 0.74 | 1.63 | 1.14 | 0.67 |
| 56 | CD22 antigen | *CD22* | NM_001771 | 0.96 | 1.05 | 1.29 | 1.30 | 1.64 | 0.96 | 0.81 |
| 57 | Keratin 15 | *KRT15* | NM_002275 | 0.98 | 0.94 | 0.81 | 1.51 | 2.08 | 0.79 | 0.82 |
| 58 | Connective tissue growth factor | *CTGF* | NM_001901 | 0.84 | 0.71 | 0.95 | 1.32 | 1.65 | 0.68 | 0.78 |
| 59 | Guanine nucleotide binding protein (G protein), beta polypeptide 2 | *GNB2* | NM_005273 | 0.73 | 0.75 | 0.55 | 1.36 | 2.05 | 0.92 | 0.68 |
| 60 | CDC-like kinase 3 | *CLK3* | NM_001292 | 0.66 | 1.00 | 0.47 | 1.37 | 1.33 | 0.80 | 0.63 |
| 61 | E2F transcription factor 1 | *E2F1* | NM_005225 | 0.77 | 0.62 | 0.62 | 2.12 | 2.22 | 1.01 | 0.52 |
| 62 | Cadherin 13, H-cadherin | *CDH13* | NM_001257 | 1.70 | 1.14 | 1.03 | 0.76 | 1.07 | 1.01 | 0.70 |
| 63 | Hemoglobin, gamma G | *HBG2* | NM_000184 | 1.52 | 1.44 | 1.02 | 1.22 | 1.05 | 0.93 | 0.63 |
| 64 | Annexin A8 | *ANXA8* | NM_001630 | 1.06 | 1.05 | 1.08 | 0.88 | 1.15 | 1.01 | 0.47 |
| 65 | Ring-box 1 | *RBX1* | NM_014248 | 0.97 | 1.19 | 0.83 | 1.02 | 0.75 | 0.97 | 0.46 |
| 66 | S100 calcium binding protein P | *S100P* | NM_005980 | 1.09 | 1.76 | 1.49 | 1.84 | 0.90 | 2.08 | 0.36 |
| 67 | Keratin 13 | *KRT13* | NM_002274 | 0.93 | 0.90 | 0.87 | 1.35 | 1.68 | 0.59 | 0.36 |
| 68 | Keratin 14 | *KRT14* | NM_000526 | 1.17 | 0.65 | 0.86 | 1.39 | 2.72 | 0.49 | 0.25 |
| 69 | A disintegrin and metalloproteinase domain 17 | *ADAM17* | NM_003183 | 0.75 | 0.71 | 0.87 | 0.29 | 0.99 | 0.50 | 0.32 |
| 70 | Ataxin 1 | *ATXN1* | NM_000332 | 0.94 | 0.87 | 0.97 | 0.90 | 1.62 | 1.04 | 0.27 |
| 71 | Protein kinase C, theta | *PRKCQ* | NM_006257 | 0.61 | 0.77 | 0.79 | 0.70 | 1.00 | 1.27 | 0.23 |
| 72 | Interleukin 1 receptor, type II | *IL1R2* | NM_173343 NM_004633 | 2.09 | 2.24 | 1.79 | 3.61 | 1.91 | 7.22 | 0.20 |
| 73 | Arachidonate 5-lipoxygenase-activating protein | *ALOX5AP* | NM_001629 | 0.30 | 1.44 | 1.63 | 0.15 | 0.21 | 0.29 | 0.66 |
| 74 | Thioredoxin-like 5 | *TXNL5* | NM_032731 | 0.29 | 0.35 | 1.20 | 2.43 | 0.36 | 0.75 | 0.72 |
| 75 | Discoidin domain receptor family, member 2 | *DDR2* | NM_006182 | 0.88 | 0.43 | 0.61 | 0.56 | 0.86 | 0.84 | 1.29 |
| 76 | Protein kinase C, eta | *PRKCH* | NM_006255 | 0.85 | 0.44 | 0.48 | 0.59 | 0.41 | 0.65 | 0.93 |
| 77 | Peroxisomal biogenesis factor 14 | *PEX14* | NM_004565 | 0.70 | 0.55 | 0.66 | 0.72 | 0.70 | 0.48 | 1.05 |
| 78 | Nedd4 family interacting protein 2 | *NDFIP2* | NM_019080 | 0.70 | 0.59 | 0.80 | 0.69 | 0.71 | 0.34 | 0.86 |

| | | | | | | | | | | |
|---|---|---|---|---|---|---|---|---|---|---|
| 79 | 3-hydroxy-3-methylglutaryl-Coenzyme A synthase 1 | *HMGCS1* | NM_002130 | 0.35 | 0.46 | 0.93 | 0.71 | 0.53 | 0.44 | 0.57 |
| 80 | Serine/threonine kinase 4 | *STK4* | NM_006282 | 0.67 | 0.62 | 0.47 | 0.89 | 0.66 | 0.65 | 0.55 |
| 81 | Myristoylated alanine-rich protein kinase C substrate | *MARCKS* | NM_002356 | 0.72 | 0.76 | 0.69 | 0.92 | 0.59 | 0.45 | 0.57 |
| 82 | Tubulin, beta 6 | *TUBB6* | NM_032525 | 0.77 | 0.79 | 0.65 | 0.81 | 0.75 | 0.57 | 0.43 |
| 83 | Fibroblast growth factor receptor 1 | *FGFR1* | NM_015850 NM_000604 NM_023105 NM_023106 NM_023107 NM_023108 NM_023109 NM_023110 NM_023111 | 0.76 | 0.30 | 0.71 | 0.66 | 0.17 | 0.57 | 0.69 |
| 84 | Thrombospondin 1 | *THBS1* | NM_003246 | 0.87 | 0.64 | 0.76 | 0.88 | 0.29 | 0.61 | 0.48 |
| 85 | Transcription factor 12 | *TCF12* | NM_003205 NM_207040 NM_207038 NM_207037 NM_207036 | 0.71 | 0.53 | 0.60 | 0.69 | 0.35 | 0.45 | 0.37 |
| 86 | NPC440-S-0223# | | | 0.97 | 0.94 | 0.68 | 0.88 | 0.35 | 0.69 | 0.90 |
| 87 | N-ethylmaleimide-sensitive factor | *NSF* | NM_006178 | 0.89 | 0.98 | 1.01 | 0.85 | 0.49 | 0.76 | 0.73 |
| 88 | Cyclin A1 | *CCNA1* | NM_003914 | 0.98 | 0.81 | 0.83 | 0.78 | 0.34 | 0.60 | 0.65 |
| 89 | Proteasome (prosome, macropain) subunit, alpha type, 3 | *PSMA3* | NM_002788 NM_152132 | 0.94 | 0.83 | 0.76 | 0.90 | 0.47 | 0.61 | 0.59 |
| 90 | Chromosome 2 open reading frame 23 | *C2orf23* | NM_022912 | 0.74 | 0.86 | 0.74 | 0.70 | 0.44 | 0.51 | 0.62 |
| 91 | Chromosome 21 open reading frame 4 | *C21orf4* | NM_006134 | 0.95 | 0.93 | 0.97 | 0.50 | 0.47 | 0.84 | 0.82 |
| 92 | TAF9-like RNA polymerase II | *TAF9L* | NM_015975 | 0.83 | 0.96 | 0.84 | 0.45 | 0.57 | 0.63 | 0.74 |
| 93 | RNA pseudouridylate synthase domain containing 4 | *RPUSD4* | NM_032795 | 0.93 | 1.26 | 1.11 | 0.49 | 0.48 | 1.25 | 0.50 |
| 94 | Arrestin domain containing 4 | *ARRDC4* | NM_183376 | 0.94 | 1.01 | 0.90 | 0.67 | 0.56 | 0.81 | 0.50 |
| 95 | Peroxisome proliferative activated receptor, gamma | *PPARG* | NM_005037 NM_015869 NM_138712 NM_138711 | 1.03 | 1.22 | 0.97 | 0.64 | 0.75 | 1.06 | 0.48 |
| 96 | Hypothetical protein FLJ11712 | *FLJ11712* | NM_024570 | 0.80 | 1.03 | 1.70 | 1.04 | 0.50 | 0.78 | 0.56 |

| | | | | | | | | | | |
|---|---|---|---|---|---|---|---|---|---|---|
| 97 | Proteasome (prosome, macropain) subunit, beta type, 1 | *PSMB1* | NM_003518 | 0.81 | 0.95 | 0.97 | 1.12 | 0.64 | 0.46 | 0.40 |
| 98 | Ring finger protein 127 | *RNF127* | NM_024778 | 0.58 | 0.77 | 1.03 | 0.87 | 0.67 | 0.93 | 0.46 |
| 99 | Methionine adenosyltransferase II, alpha | *MAT2A* | NM_005911 | 0.62 | 0.95 | 1.01 | 0.92 | 0.77 | 0.76 | 0.28 |
| 100 | Insulin-like growth factor binding protein 5 | *IGFBP5* | NM_000599 | 0.35 | 0.31 | 0.34 | 0.81 | 0.29 | 0.22 | 1.14 |
| 101 | Insulin-like growth factor binding protein 5 | *IGFBP5* | NM_000599 | 0.43 | 0.23 | 0.22 | 0.86 | 0.31 | 0.19 | 1.14 |
| 102 | Protein kinase C, alpha | *PRKCA* | NM_002737 | 0.52 | 0.12 | 0.41 | 0.49 | 0.32 | 0.28 | 0.56 |
| 103 | SMAD, mothers against DPP homolog 7 | *SMAD7* | NM_005904 | 0.57 | 0.31 | 0.51 | 0.92 | 0.25 | 0.20 | 0.53 |
| 104 | Nucleoporin 88kDa | *NUP88* | NM_002532 | 0.44 | 0.31 | 0.42 | 0.56 | 0.41 | 0.40 | 0.45 |
| 105 | Myosin, light polypeptide kinase | *MYLK* | NM_005965<br>NM_053032<br>NM_053031<br>NM_053030<br>NM_053029<br>NM_053028<br>NM_053027<br>NM_053026<br>NM_053025 | 0.38 | 0.31 | 0.32 | 0.73 | 0.33 | 0.33 | 0.38 |
| 106 | GATA binding protein 6 | *GATA6* | NM_005257 | 0.17 | 0.15 | 0.32 | 0.98 | 0.33 | 0.35 | 0.44 |
| 107 | Matrix metalloproteinase 2 | *MMP2* | NM_004530 | 0.53 | 0.22 | 0.29 | 0.36 | 0.05 | 0.05 | 0.42 |
| 108 | Chemokine (C-C motif) ligand 2 | *CCL2* | NM_002982 | 0.38 | 0.45 | 0.40 | 1.13 | 0.12 | 0.17 | 0.20 |
| 109 | Protease, serine, 11 (IGF binding) | *PRSS11* | NM_002775 | 0.51 | 0.35 | 0.33 | 0.64 | 0.59 | 0.14 | 0.20 |
| 110 | Protease, serine, 11 (IGF binding) | *PRSS11* | NM_002775 | 0.57 | 0.32 | 0.35 | 0.56 | 0.46 | 0.11 | 0.15 |
| 111 | OC0717-S-0504# | | | 0.15 | 0.05 | 0.15 | 0.35 | 0.10 | 0.09 | 0.41 |
| 112 | Collagen, type I, alpha 1 | *COL1A1* | NM_000088 | 0.17 | 0.08 | 0.18 | 0.46 | 0.07 | 0.11 | 0.32 |
| 113 | Integrin, beta 3 | *ITGB3* | NM_000212 | 0.06 | 0.06 | 0.12 | 0.46 | 0.09 | 0.06 | 0.28 |
| 114 | Connexin 43 | *GJA1* | NM_000165 | 0.10 | 0.06 | 0.12 | 0.51 | 0.32 | 0.08 | 0.28 |

*The data shown are the ratios of the expression in arsenical-treated HUC-1 or MC-T2 cells compared to that in untreated HUC-1 cells. The ratios are the mean values for 4 independent experiments.

**Gene names and symbols are from Unigene (http://www.ncbi.nlm.nih.gov/entrez/query.fcgi? db=unigene), and sequence IDs are from Cancer Genomic Anatomy Project (http://cgap.nci.nih.gov/Genes/BatchGeneFinder).

# Indicates the clone ID from original subtraction library.
